# Differences in Neurocognitive Mechanisms Underlying the Processing of Center-Embedded and Non–embedded Musical Structures

**DOI:** 10.3389/fnhum.2018.00425

**Published:** 2018-10-23

**Authors:** Xie Ma, Nai Ding, Yun Tao, Yu Fang Yang

**Affiliations:** ^1^Institute of Psychology, Chinese Academy of Sciences, Beijing, China; ^2^College of Educational Science and Management, Yunnan Normal University, Kunming, China; ^3^Key Laboratory of Educational Informatization for Nationalities, Yunnan Normal University, Kunming, China; ^4^College of Biomedical Engineering and Instrument Sciences, Zhejiang University, Hangzhou, China; ^5^Key Laboratory for Biomedical Engineering of Ministry of Education, Zhejiang University, Hangzhou, China; ^6^State Key Laboratory of Industrial Control Technology, Zhejiang University, Hangzhou, China

**Keywords:** center-embedded structure, non-embedded structure, non-experts, experts, electroencephalography

## Abstract

In music, chords are organized into hierarchical structures based on recursive or embedded syntax. How the brain extracts recursive grammar is a central question in musical cognition and other cognitive neuroscience, but the precise mechanism remains unclear. By analyzing event related potentials (ERPs) and neural oscillatory activity, the present study investigated neurocognitive mechanisms underlying the processing of center-embedded structure in music by examining the differences in center-embedded and non-embedded structure processing and evaluating how these differences are affected by musical proficiency. Based on Western musical proficiency, the subjects were divided into two groups, non-experts and experts. The results revealed that for non-experts, the processing of center-embedded structure elicited greater early right-anterior negativity (ERAN) and N5 components as well as, reduced alpha and gamma activities than did the non-embedded structure. For experts, no significant difference in the ERP response was observed between the processing of non-embedded and center-embedded structures; however, the processing of center-embedded structure elicited increased beta activity compared to non-embedded structure. These findings indicate that listeners different in proficiency would rely on different cognitive neural mechanisms in music processing with the syntactic complexity increases.

## Introduction

The Western music system is characterized by syntactic structures based on tonal harmony relationships. As stated by both the generative theory of tonal music (GTTM; Lerdahl and Jackendoff, 1987) and the generative syntax model (GSM; Rohrmeier, 2011), the organized structure of harmonic syntax strongly resembles that of language (Lerdahl and Jackendoff, 1987; Patel, 2003; Rohrmeier, 2011). It exceeds the simplicity of a straightforward and linear chord transition order (Chomsky, 1956; Rohrmeier and Cross, 2009; Rohrmeier, 2011), and can be modeled by higher-level and recursive grammar, termed phrase structure grammar, which can generate a hierarchical structure by embedding sub-structures in its own center (Rohrmeier, 2011; Rohrmeier and Rebuschat, 2012; Rohrmeier et al., 2014). In language, the sentence “The teacher (that the boy saw) persistently tries” is formed by center-embedding “the boy saw” into “The teacher persistently tries.” A hallmark of processing such a center-embedded sentence is that learners need to attach the matrix clause verb “tries” to the initial noun-phrase “The teacher,” involving a syntactic long-distance dependency. Similarly, the formation of the musical sentence “key 1-key 2-key 1,” is formed by center-embedding “key 2” on top of “key 1” at higher-level. To process such center-embedded structure, learners need to integrate the separate syntactic dependency in “key 1” harmonic context (Rohrmeier and Cross, 2009; Koelsch et al., 2013; Woolhouse et al., 2015).

The acquisition and processing of recursive or center-embedded structure is a difficult and complicated cognitive process, as revealed by studies in the field of language and other cognitive disciplines. Developmental studies have revealed that although children grasp basic and simple syntax with surprising speed, they cannot exactly grasp non-adjacent syntax involving a center-embedded structure until the age of 7 years (Schipke et al., 2012; Fengler et al., 2015; Skeide and Friederici, 2016). Studies directly comparing the online processing of center-embedded vs. non-embedded sentences have found that processing of the verbs in matrix clauses marking the complication of long-distance dependency in embedded sentences is more difficult than processing the verb in non-embedded sentences, indicated by slower reaction times (Stromswold et al., 1996), lower accuracy rate (Opitz and Friederici, 2007) and larger ERPs, such as LAN or P600 (Vos et al., 2001; Phillips et al., 2005; Li and Zhou, 2010).

Since the acquisition and processing of sequences with center-embedded structure are more complicated than those without recursive or embedded structure, it is suggested that cognitive processes and neural mechanisms underlying the processing of two syntactic structures must be different (Gibson, 1998; Christiansen and Chater, 2003; Badre, 2008; Friederici and Singer, 2015; Jeon and Friederici, 2015). Many studies have demonstrated that, compared to the non-embedded structure, processing of embedded or recursive structures may result in an increased demand for the syntactic unification required by more complex, higher-order mental representations (Phillips et al., 2005; Friederici, 2011; Friederici and Singer, 2015; Skeide and Friederici, 2016). For example, converging evidence from imaging studies revealed that when processing the verb in matrix clause, the increase in the number of embedded clauses, increased the activation of BA 44, the posterior region of the inferior frontal gyrus (Opitz and Friederici, 2007; Makuuchi et al., 2009; Jeon and Friederici, 2013), that is part of the neural circuit responsible for processing complex grammatical structures and it has been viewed as the system that supports syntactic computation, reanalysis and reconstruction driven by top-down processing (Friederici, 2006, 2011; Brouwer and Hoeks, 2013). Furthermore, by analyzing neural oscillatory activity, some studies found that beta power was higher for main verbs in center-embedded relative clauses compared to non-center-embedded relative clauses (Weiss et al., 2005). Higher beta power may be related to a greater demand on syntactic integration, indicating a syntactic reanalysis and repairing process relying on abstract structural knowledge activation (Bastiaansen and Hagoort, 2006, 2015; Meyer et al., 2013; Lewis et al., 2015, 2016a).

The differences in the cognitive and neural mechanisms underlying center-embedded and non-embedded structure processing are also reflected in the involvement of general cognitive mechanisms such as cognitive control, including attention, working memory, monitoring and inhibition (Gibson, 1998; Gibson and Thomas, 1999; Vos et al., 2001; Badre, 2008; Makuuchi and Friederici, 2013; Jeon and Friederici, 2015). For example, Vos et al. (2001) found that the processing of the violation of main verbs in center-embedded sentences induced greater right-anterior negativity (LAN) than the processing of verbs in conjoined sentences; meanwhile, the effect interacted with individual working memory capacity, implying that the difficulties in embedded structure processing could be partly ascribed to the higher working memory load (Gibson, 1998). Many imaging studies have found that, compared to non-embedded sequences, the processing of main verbs in center-embedded sequences elicited activation of more anterior portion of the prefrontal cortex (PFC; e.g., BA 10), which is thought to be associated with cognitive control (Jeon and Friederici, 2013, 2015; Friederici and Singer, 2015).

Studies on musical syntax cognition have primarily focused on the processing of local or non-embedded syntactic structure. It was found that, when a target chord was syntactically less-related or less-expected to the harmonic contexts, two ERP components, early right-anterior negativity (ERAN) in the time window of approximately 150–300 ms, and N5 in the time window of approximately 500–800 ms, were typically evoked (Koelsch and Jentschke, 2009; Koelsch et al., 2013). The two components have been taken as the indexes of the degree of consistency of an incoming chord to the contextual syntactic constraint (Koelsch et al., 2000; Poulin-Charronnat et al., 2006; Koelsch and Jentschke, 2009; Koelsch et al., 2013). Comparatively, fewer studies have explored the integration of complex recursive or center-embedded structure in music (Tillmann et al., 1998; Rohrmeier and Cross, 2009; Koelsch et al., 2013; Woolhouse et al., 2015). Until recently, Koelsch et al. (2013) investigated the ERP patterns corresponding to hierarchical (center-embedded) structure processing, and found that the syntactically less-related relationship in long-distance dependencies between the final chords and the first phrase induced greater ERAN and N5 components, and their time windows were not significantly different from those in response to non-embedded structures, as found in previous studies (Poulin-Charronnat et al., 2006; Koelsch and Jentschke, 2009). Ma et al. (in press) explored the processing of non-embedded and center-embedded harmonic structures for Chinese subjects, and found that in the non-embedded condition, the less-related final chords evoked ERAN-N5 biphasic responses in moderate- and high-proficiency groups; in the center-embedded condition, however, only the high-proficiency group showed an ERAN-N5 biphasic response. This result indicated that relative to non-embedded structure, the sensibility of center-embedded structure could be acquired through more training. However, these studies did not directly explore the neurocognitive mechanisms during center-embedded musical structure processing, specifically, whether the neurocognitive mechanisms involved in processing center-embedded structure are distinct from those involved in processing non-embedded structure and what differences there are between the two structure processing remain unclear.

Using ERPs and oscillatory neural activity, the present study directly compared the cognitive and neural oscillatory mechanisms underlying the processing of two kinds of musical syntactic structures, center-embedded and non-embedded structures. Two type of syntactic sequences differing in complexity were used in this study: a simple structure without modulation or transposition (without a key change from the original key to another) was defined as a non-embedded structure, whereas a complex structure with modulation or transposition (with a key change) in the center of the sequence was defined as a center-embedded and recursive structure according to the GSM and GTTM (Lerdahl and Jackendoff, 1987; Rohrmeier and Cross, 2009; Rohrmeier, 2011; Koelsch et al., 2013; Woolhouse et al., 2015). The center-embedded sequences were selected from previous studies (Koelsch et al., 2013), and the non-embedded sequences were obtained by modifying the same center-embedded sequences. In these two type of sequences, the final tonic chords closed the dominant chords of the first phrase ending, which forms a syntactic long-distance dependency at the upper level (the dominant function has the role of creating instability that requires tonic for resolution and it is a basic rule in harmonic syntax). However, in the center-embedded sequences, a lower level segment of a local key (modulation or transposition) was nested into a higher level segment of an overarching key; whereas in the non-embedded sequences, no modulation was nested in and all segments maintained in a single key.

We first aimed to determine whether processing musical sequences with center-embedded structure were more difficult than that with non-embedded structure, as reflected by the response to the final chords completing the long-distance dependency. For the increased cost caused by the center-embedded structure in language processing, some psycholinguists have proposed that integrating the detached main clause with long-distance dependencies required for that the early syntactic information of main clause be kept active in working memory until the thematic roles were assigned (find the verbs in matrix clauses; Gibson, 1998; Vos et al., 2001; Phillips et al., 2005). However, compared to non-embedded sentences where no argument structure (NP+V) is embedded, the processing of an argument structure of a subordinate clause in center-embedded sentences, would suppress or interfere the activation of early syntactic representation, and hindering the attachment of syntactic long-distance dependencies (Gernsbacher, 1997; Gibson and Thomas, 1999; Kaan et al., 2000; Lewis et al., 2006). We considered that such increased cost might also occur during the processing of musical center-embedded structures. To integrate long-distance dependencies in musical structure, the initial tonal syntactic representation needs to be kept active in working memory as soon as possible (Tillmann et al., 1998; Koelsch et al., 2013). Nevertheless, processing of the nested new tonal representations (modulation/transposition) in center-embedded structure would suppress or interfere with the activation of early tonal syntactic representation (Gernsbacher, 1997; Gibson and Thomas, 1999; Kaan et al., 2000; Lewis et al., 2006) and reducing the perceptual consistency of the detached structure at the upper level. Therefore, it was our hypothesis that although the final tonic chords perfectly closed the dominant chords of the first phrase ending with long-distance dependencies in both center-embedded and non-embedded structures, the nested transition or modulation might interfere with the construction of long-distance dependencies. The effect could be reflected by the difficulty of the final chords processing in the center-embedded sequences indicated by a larger ERAN or N5 than in the non-embedded sequences.

The effect of musical experience and proficiency of individuals should be considered. Studies on linguistic syntactic processing have revealed that the processing difficulties arising from syntactic complexity were more prominent for low-proficiency individuals than high-proficiency individuals (King and Kutas, 1995; Clahsen and Felser, 2006; Jackson and van Hell, 2011). High-proficiency individuals with a higher sensitivity of syntax could effectively suppress the interference of surface structure and construct deep syntactic representations with a lower processing cost in complex sentences processing (King and Kutas, 1995; Gibson, 1998; Clahsen and Felser, 2006; Jackson and van Hell, 2011). In music domain, the advantage of musicians with higher-proficiency in their sensitivity to syntactic structure compared to non-musicians with lower-proficiency have been found widely in harmonic syntax processing (Koelsch et al., 2002; Poulin-Charronnat et al., 2006). Could the high-proficiency individuals are capable of quickly attachment the syntactic long-distance dependencies in complex center-embedded sequences in music, so that have less difficulty processing center-embedded structure compared to simple non-embedded structure? In total, we would investigate the difference in ERP responses in the processing of musical sequences with center-embedded and non-embedded structure, and investigate the influence of the listeners’ musical proficiency. Two groups of Chinese listeners varying in Western musical proficiency were recruited, with the expert group receiving formal training of western music at least for 10 years, and the non-expert group with no professional training but listening to Western music at leisure. We hypothesized that for the non-experts, the difficulty in processing center-embedded structure may be significant, as reflected by processing of the final chords in center-embedded sequences may evoke a larger ERAN or N5 than that in non-embedded sequences; whereas for the experts, the difficulty in processing center-embedded structure may not be significant and no ERAN or N5 is elicited in processing of the final chords in center-embedded sequences than that in non-embedded sequences.

Additionally, we explore the different neural oscillatory mechanisms underlying the processing of these two syntactic structures using EEG time-frequency (TF) analysis. Oscillatory activity (non-phase-locked) in the magnetoencephalography/EEG signals can offer complementary or additional information regarding the neurocognitive mechanisms (Tallon-Baudry and Bertrand, 1999; Bastiaansen et al., 2012). For example, in the literature, it is suggested that neural oscillatory activity in the beta band (13–30 Hz) reflects the active maintenance of/changes in the underlying neurocognitive network responsible for the representation and construction of the present sentence meaning (Bressler and Richter, 2015; Lewis et al., 2015, 2016b). There appears to be a link between changes in beta power and both the manipulation of syntactic unification in language sentence processing (Weiss et al., 2005; Bastiaansen and Hagoort, 2006; Meyer et al., 2013; Schneider et al., 2016) and musical sentence processing (Akrami and Moghimi, 2017). The higher beta power is generally related to more demanding syntactic parsing and integration driven by top-down processing (Bressler and Richter, 2015; Lewis et al., 2015, 2016b). A change in the power of alpha frequency range generally correlates with cognitive control (such as selective attention and sustained alertness), indicating the involvement of general cognitive resources other than the domain-specific mechanism (Meyer et al., 2013; Sadaghiani and Kleinschmidt, 2016). A decrease in alpha power can be related to an increased demand on cognitive control (Ruiz et al., 2009; Wang et al., 2012a; Wilsch et al., 2015; Sadaghiani and Kleinschmidt, 2016; Rommers et al., 2017).

Many studies have provided evidences demonstrating that the system used for “online structural integration” in music and language may be shared based on the syntactic integration resource hypothesis (Patel, 2003; Fedorenko et al., 2009; Jeon, 2014; Farbood et al., 2015). We postulate that hierarchical structure processing in music may show some commonalities with hierarchical structure processing in language; that is, the processing of center-embedded musical structure, compared with non-embedded musical structure, may evoke different patterns of neural oscillations activity, especially on beta and alpha frequency bands that reflect the involvements of structural unification mechanism and general cognitive control function, respectively.

Individuals with different proficiency have been found to use different processing mechanisms during the musical syntax processing. For example, a number of studies have demonstrated that the Broca’s area (BA44 and BA 45) was more engaged in processing musical syntax for the high-proficiency listeners than for the low-proficiency listeners (Koelsch et al., 2005; Wehrum et al., 2011; Wakita, 2014). Linguistic syntactic researches have directly compared different mechanisms involved in low- and high-proficiency systems processing in response to increased syntactic complexity. Jeon and Friederici (2013) found that in the native language sentence with high-proficiency, the recursive structure processing induced enhanced activation mainly in the posterior region of BA44 as hierarchies become more complex, whereas second language and non-language sequences with low-proficiency processing evoked activation in more anterior portions of the PFC (such as BA10/47). It was suggested that, in order to cope with more complicated recursive grammar, the processing of high-proficiency system was primarily dependent on “structural unification” mechanisms driven by top-down processing; however, the processing of low-proficiency system may recruit more general cognitive mechanisms. Hence, we further hypothesized that the difference in processing mechanisms for center-embedded and non-embedded structure in music would be affected by proficiency. For the experts, the processing of the final chords in center-embedded sequences may elicit a larger beta power than that in non-embedded sequences, indicating that they may rely more on the top-down unification mechanism when the syntactic complexity increased. For the non-expert group, the processing of the final chords in center-embedded sequences may induce decreased alpha activity than that in non-embedded sequences due to their more reliance on general cognitive control function.

## Materials and Methods

### Participants

All participants were from Yunnan Normal University and Yunnan Arts University, were born and raised in China (age range 20–24 years, mean = 22.9 years), and were without abnormal hearing or absolute pitch. We recruited 18 experts with high proficiency in Western tonal music (10 female, eight male), and 18 non-experts with a lower proficiency level (nine female, nine male). The expert group had ≥10 years of professional music training in Western instrument performance such as piano or violin. The non-expert group received no formal music training; however, they listened to Western music, including Western pop and classical music. This study was approved by the local ethics committee of Yunnan Normal University, and all participants provided written informed consent. This study conforms to the ethical principles of the Declaration of Helsinki (World Medical Organization, 1996).

### Stimuli

**Figure 1:**
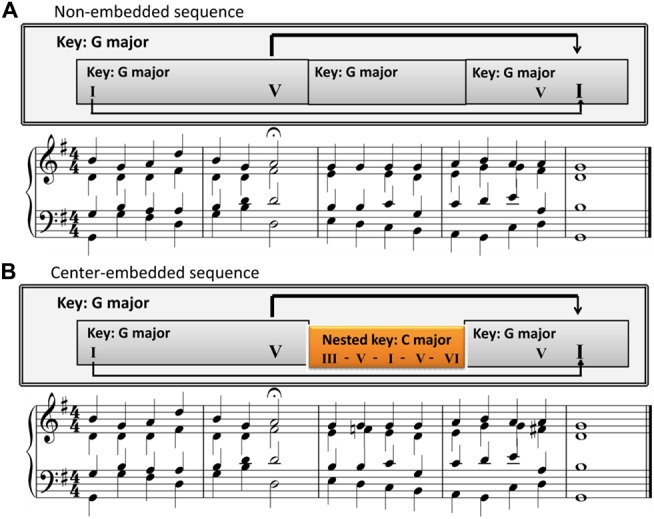
A sample excerpt illustrating the two sequences with different structures.** (A)** The non-embedded sequences. Chord sequences without transition or modulation segments. All parts are in G major. **(B)** The center- embedded sequences. Chord sequences with transition or modulation segments. The C major segment is nested into an overarching key of G major.

The center-embedded sequences were based on those used in Koelsch et al. (2013). The first excerpt was selected from BWV 373 by J. S. Bach, while the second excerpt was from BWV 302 by J. S. Bach. The structure of for both musical excerpts was center-embedded. The first phrase ended with a half cadence on the dominant, and the second phrase progressed in another key (known as a transition or modulation), and concluded with the initial tonic through an authentic cadence (a progression from the dominant chord to the tonic chord that perfectly concludes a phrase, section, or piece of music). It featured a lower level segment in a local key, which was embedded in a higher-level segment in an overarching key (Figure 1B and Supplementary Figure S1B). With regard to the GTTM and GSM (Lerdahl and Jackendoff, 1987; Rohrmeier, 2011), the final chord of the excerpt prolonged the first chord of the first phrase and closed the dominant of the first phrase ending. In addition, a modulation was nested into the middle between the first phrase and the final chord (see the arrows in Figure 1B); therefore, the structure was nested and center-embedded (Rohrmeier, 2011; Rohrmeier and Rebuschat, 2012; Rohrmeier et al., 2014). Previous studies suggested that there was an interaction between temporal structure and syntactic structure in music processing and the syntactic integration was influenced by temporal structure (Bigand et al., 1999; Tillmann and Lebrun-Guillaud, 2006). In order to remove the interferences from rhythm, the main harmonic structure (function) was extracted from the original pieces, which retained the main syntactic component and removed the decorated component. The music stimulus was presented with a consistent rhythm by a quarter note by reference to previous research (Rohrmeier and Cross, 2009; Woolhouse et al., 2015; Figure 1B and Supplementary Figure S1B).

The non-embedded sequences were obtained by modifying the center-embedded sequences based on previous studies (Bigand et al., 1999; Woolhouse et al., 2015; Figure 1A and Supplementary Figure S1A). Since the long-distance dependencies in the center-embedded sequence may generate additional memory load for sequence processing (Makuuchi et al., 2009; Makuuchi and Friederici, 2013; Meyer et al., 2013), to reduce this effect, we also maintain the long-distance dependency by detaching the dominant chord and tonic chord. The first phrase remained unchanged and ended with a half cadence on the dominant. The second phrase continued in the same key, but did not begin with the tonic. The ending of the second phrase was still at the initial tonic, which also remained unchanged compared to the embedded sequence (Figure 1A and Supplementary Figure S1A). In this sequence, the final chord also prolonged the first chord of the first phrase and closed the dominant of the first phrase ending (see the arrows in Figure 1A and Supplementary Figure S1A), but no modulation was nested in the middle between the first phrase and the final chord (each segment was in a single key).

The center-embedded musical sequence was similar to the center-embedded English sentence “The teacher (that the boy saw) persistently tries”; whereas the non-embedded musical sequence was similar to the non-embedded English sentence “The teacher (in a blue coat) persistently tries.” The dominant of the first phrase ending can be analogized to the matrix clause NP “The teacher” in the center-embedded sentence, and also to the NP “The teacher” in the non-embedded sentence. The final chord can be analogized to the matrix clause verb “tries” in the center-embedded sentence, and also to the verb “tries” in the non-embedded sentence as all of them are the core syntactic components, and serve the function of ending (completing) the entire long-distance dependency grammar. ERP and oscillatory activity responses to the final chord can reveal the construction of a syntactic long-distance dependency with and without an embedded segment (transition or modulation) added.

Using Sibelius 6.2 software, each stimulus at each condition was turned into six major keys, after which the files were rendered using a piano sound at a tempo of 100 beats per minute and archived in wav format. The rendering processes yielded 24 stimuli: 6 keys × 2 excerpts × 2 structures. Additionally, 16 other stimuli were created with one chord played with bassoon sound, rather than piano sound. These timbre deviants were spread equally in different positions (4) and different conditions (2 excerpts × 2 structures). The 16 stimuli containing timbre deviants were not used in the analysis of ERPs; but were used for a timbre detection task.

### Procedure

This procedure was designed with reference to previous studies (Koelsch and Jentschke, 2009; Koelsch et al., 2013). The EEG recording session involved the participants watching a muted video without captions while playing the stimuli at 60 dB sound pressure level (SPL). All 24 stimuli without timbre deviants were presented five times in random order while the 16 stimuli with timbre variations were presented only once. They were randomly intermixed across the experiment, amounting to 136 sequences in total. The subjects were instructed to monitor filtered chords (timbre variations chords) and to identify them by clicking a response button. A total of 136 sequences were played for duration of approximately 40 min. After the recording, the participants were instructed to assess (conclusiveness rating) eight stimuli (2 keys × 2 excerpts × 2 structures), and answer the question “How well did the last chord end the whole sequence?” on a scale of 1 (not at all) to 9 (perfectly ended).

### EEG Recordings and Data Analysis

#### ERP Analysis

Brain electrical activity was recorded via EEG (Brain Products, Munich, Germany) using 64 Ag/AgCl electrodes mounted on an elastic cap. The data were referenced online to FCz and offline re-reference analysis to the algebraic average of the left and right mastoids (Luck, 2005). A continuous sampling rate of 1,000 Hz and a finite impulse response (FIR) filter (0.05–100 Hz band filter) were used to amplify the signals for offline analysis. The horizontal electrooculograms (EOGs) and vertical EOGs were collected from the left and the right orbital rims and infra- and supra-arbitrarily at the left eye, respectively. The impedance of the EOG electrodes was maintained at >5 kΩ.

Pre-processing of raw EEG data was performed using Brain Vision Analyzer 1.05 (Brain Products, Munich, Germany). Initially, ocular artifacts were corrected, and data were filtered offline with a band-pass filter of 0.1–25 Hz (24 dB/octave). The data were then segmented from −200 ms to 1,200 ms after the onset of the final chord, with baseline correction from 200 ms to 0 ms preceding the chord onset. Next, based on the eye movement correction algorithm (Gratton et al., 1983), eye movements and blinks (EOG artifacts) were corrected. Offline computerized artifact rejection (mean EOG voltage exceeding ±80 μV) was applied. In addition, trials containing artifacts caused by muscle movements or amplifier block were removed using the same threshold. Referring to the study by Koelsch et al. (2013), the ERPs elicited by the final chords in different conditions were marked and analyzed. For statistical analysis of the ERPs, 10 regions of interest (ROIs) were computed: left frontal (AF3, F1, F3, F5); left frontal central (FC1, FC3, FC5); left central (C1, C3, C5); left central parietal (CP1, CP3, CP5); left parietal (P1, P3, P5, PO3); right frontal (AF4, F2, F4, F6); right frontal central (FC2, FC4, FC6); right central (C2, C4, C6); right central parietal (CP2, CP4, CP6); and right parietal (P2, P4, P6, PO4).

ERPs were conducted statistically by repeated-measures ANOVAs, treating factor Western tonal musical proficiency group (experts, non-experts) as a between -participant variable, treating the factors of syntactic structure (non-embedded, embedded), hemisphere (left and right ROIs), and anterior–posterior distribution (frontal, frontal central, central, central parietal and parietal ROIs) as within-participant variables. Only the significant or marginally significant effects of structure and the significant interactions involving structure were reported. For all analyses, the degrees of freedom of the *F* ratio were corrected for violations of the sphericity assumption based on Greenhouse-Geisser correction (Greenhouse and Geisser, 1959), and Bonferroni corrections were used for each comparison (Keppel, 1991).

#### Time Frequency Analysis

The fieldtrip software package, which is an open-source MATLAB toolbox for neurophysiological data analysis, was used to perform TF analyses (Oostenveld et al., 2011). In the pre-processing stage, the EEG data were segmented into 3 s epochs ranging from −1 s to 2 s relative to the onset of the final chords and were referenced online to FCz and offline re-reference analyzed to the algebraic average of the left and right mastoids (Luck, 2005). Trials with apparent artifacts were then deleted based on visual inspection of the trials. Independent component analysis (ICA) was then performed (Bell and Sejnowski, 1995; Jung et al., 2000) to eliminate such components associated with the ocular artifacts. In the TF analysis, as a method of optimizing the trade-off between frequency resolution and time, the Fourier spectra of single trials (segmented from −500 ms to 1,500 ms relative to the onset of the final chord) were calculated for each participant using two different methods based on different frequency ranges. In the low frequency range (2–30 Hz), a 500 ms time-smoothing window with a Hanning taper was utilized in time steps of 50 ms and frequency steps of 2 Hz. In the high frequency range (25–100 Hz), a 400 ms time-smoothing window and a 5 Hz frequency-smoothing window with a multi-taper approach were utilized in time steps of 50 ms and frequency steps of 2.5 Hz. The power spectra of single trials were calculated and averaged under each condition (non-embedded, center-embedded) for each subject. The outcome of subject-average power variation in the post-stimulus time range was expressed as a relative variation from the baseline time range (from −350 ms to −50 ms prior to the onset of the final chord), independently for each condition. The average TF representation of power over all participants and scalp electrodes was then calculated based on visual inspection.

The statistical analysis of the TF responses was performed using a cluster-based random permutation test as described by Maris and Oostenveld (2007). For each data point of the two conditions (electrode by frequency and by time), a simple dependent-sample *t*-test was performed. All adjacent data points above the significance level (5%) were categorized into clusters. Cluster-level statistics were calculated by taking the sum of the t-values for each cluster. A null distribution that assumed no difference between the conditions was then established. This null distribution is obtained by 1,000 times randomly assigning the conditions in subjects and calculating the largest cluster-level statistic for each randomization. Lastly, the observed cluster-level test statistics between two conditions are compared against the null distribution. The clusters falling in the highest or lowest 2.5% confidence interval are considered significant (Maris and Oostenveld, 2007; Oostenveld et al., 2011).

The specific analytical procedure was designed by consulting the previous researches (Wang et al., 2012b; Bastiaansen and Hagoort, 2015; Lewis et al., 2016a). The permutation tests for examining a potential effect between the two structures (non-embedded, center-embedded) were applied to the time window 0–1,200 ms after final chord onset separately for low frequency ranges (2–30 Hz) and high frequency ranges (30–100 Hz). Further, the mean power values of each condition were compared in the selected frequency window and time window based on the results of the permutation tests and visual inspection.

## Results

### ERP Data

**Figure 2:**
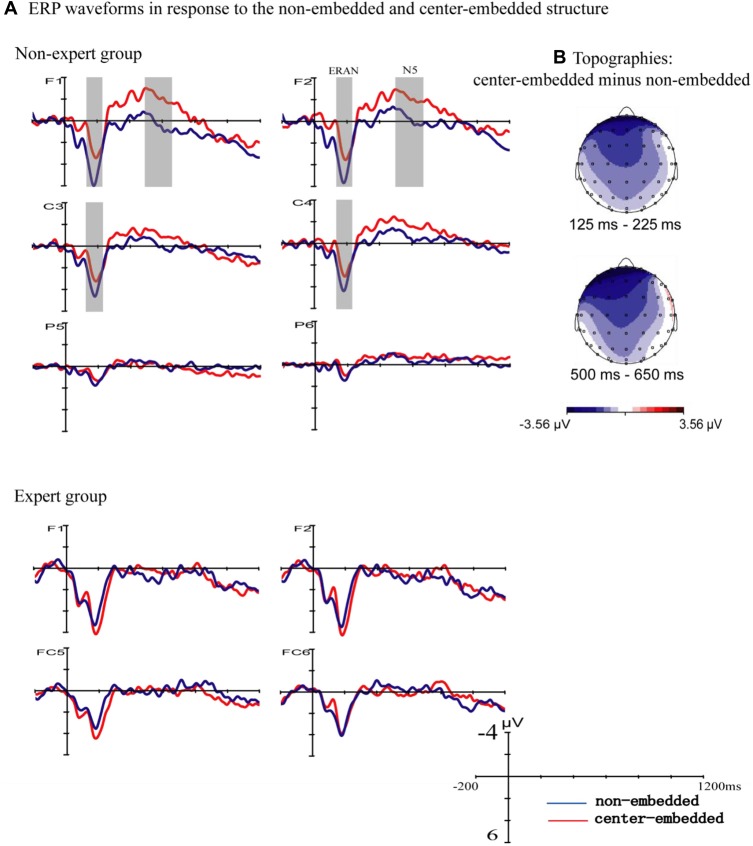
Event related potentials (ERPs) to the final chords with the two structures. **(A)** The ERPs evoked by the final chords in the non-embedded and center-embedded conditions are presented separately for the non-expert and expert groups. In the non-expert group, compared to the ERPs evoked by the non-embedded condition (blue waveforms), the center-embedded condition (red waveforms) evoked an early negativity (around 125–225 ms), and a later negativity (around 500–650 ms). The gray shaded areas reflect a statistically significant difference between the two conditions. In the expert group, the ERP effects did not significantly differ between the two conditions. **(B)** The scalp distribution of the early and late ERP effects elicited by the final chords of different conditions (difference potentials: center-embedded minus non-embedded) in the non-expert group.

Figure 2 presents the ERP responses to the final chords of non-embedded and center-embedded sequences. In the non-expert group, compared to the non-embedded condition, the final chords of the center-embedded condition evoked early negative potentials with a bilateral frontal-central scalp distribution (approximately 125–225 ms after the onset of the final chord) that emerged in the standard ERAN latency range (Koelsch and Jentschke, 2009; Koelsch et al., 2013), followed by a later frontal negativity (approximately 500–650 ms after the onset of the final chord) that emerged in the standard N5 latency range (Poulin-Charronnat et al., 2006; Koelsch and Jentschke, 2009); however, these effects were not clearly observed in the expert group.

A repeated-measure ANOVA using group (non-experts, experts) as a between-subjects factor, and syntactic structure (non-embedded, center-embedded), hemisphere (left, right ROIs), and anterior-posterior distribution (frontal, frontal central, central, central parietal and parietal ROIs) as the within-subjects factors was carried out for the time window of 125–225 ms. The results indicated an interaction effect between structure and group (*F*_(1,34)_ = 6.76, *p* = 0.014, $\eta_{\text{p}}^{2}$ = 0.17) and a significant interaction effect among structure, anterior-posterior distribution, and group based on the Greenhouse-Geisser correction (*F*_(1.42,42.894)_ = 3.65, *p* = 0.048, $\eta_{\text{p}}^{2}$ = 0.10). Simple effect analyses reflected that, for the non-expert group, the negativity was larger for the center-embedded condition than for the non-embedded condition (*F*_(1,34)_ = 9.83, *p* = 0.004, $\eta_{\text{p}}^{2}$ = 0.224), and the effect was only statistically significant in the frontal (*p* = 0.002), frontal central (*p* = 0.004), central (*p* = 0.018), and central parietal (*p* = 0.015) regions; for the expert group, in contrast, the difference between the non-embedded and center-embedded conditions was not significant.

The analogous ANOVA for a later time window (500–650 ms) showed a marginally non-significant interaction effect for syntactic structure, anterior-posterior distribution, and group based on the Greenhouse-Geisser correction (*F*_(1.627,55.322)_ = 3.10, *p* = 0.063, $\eta_{\text{p}}^{2}$ = 0.084). Simple effect analyses revealed that in the non-expert group, negativity was larger in the center-embedded condition than in the non-embedded condition (*F*_(1,34)_ = 5.39, *p* = 0.026, $\eta_{\text{p}}^{2}$ = 0.14), and was more prominent at the frontal (*p* = 0.002) and frontal central (*p* = 0.018) regions. However, no main effect or interaction effect of structure was found.

### TF Data

**Figure 3:**
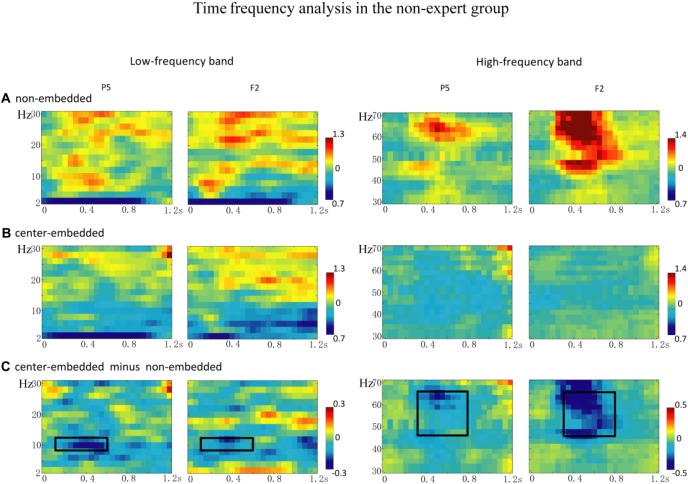
The time frequency analysis of final chords in the non-expert group.** (A,B)** Time frequency representations of the non-embedded and center-embedded conditions at electrodes P5 and F2 for the low and high frequency ranges. **(C)** Contrasts of the center-embedded and non-embedded conditions at the same electrodes. The black boxes indicate that, at low frequency, the alpha-band power (8–12 Hz) within the selected time window (100–600 ms) was significantly different between the two conditions; at high frequency, the gamma-band power (45–65 Hz) within the selected time window (300–800 ms) was significantly different between the two conditions.

**Figure 4:**
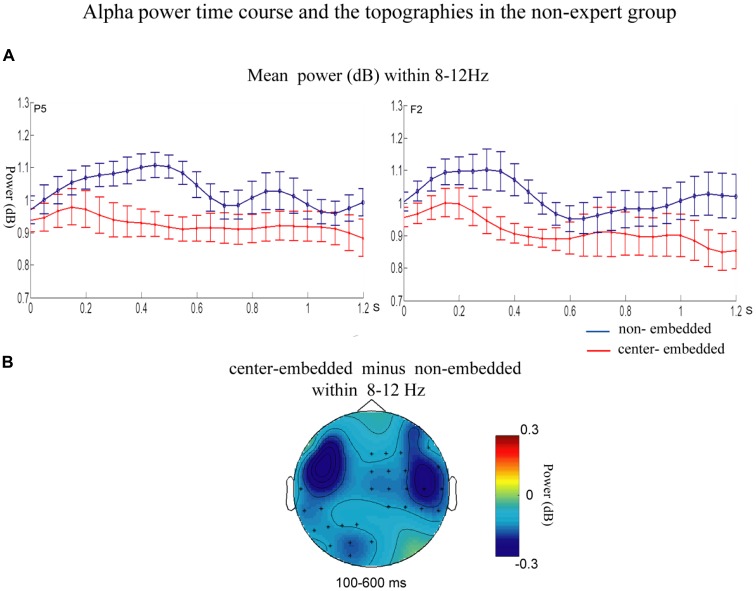
Time course of the mean alpha power and difference topographies of final chords in the non-expert group.** (A)** Temporal course of the mean alpha power (8–12 Hz) between 0 and 1200 ms after the onset of the final chords for the non-embedded and center-embedded conditions at electrodes P5 and F2. Error bars indicate standard errors. **(B)** The difference topographies (center-embedded minus embedded) in the alpha band (8–12 Hz) within the 100–600 ms. The electrodes that showed significant effects over 30% of the selected time intervals were marked by asterisks.

**Figure 5:**
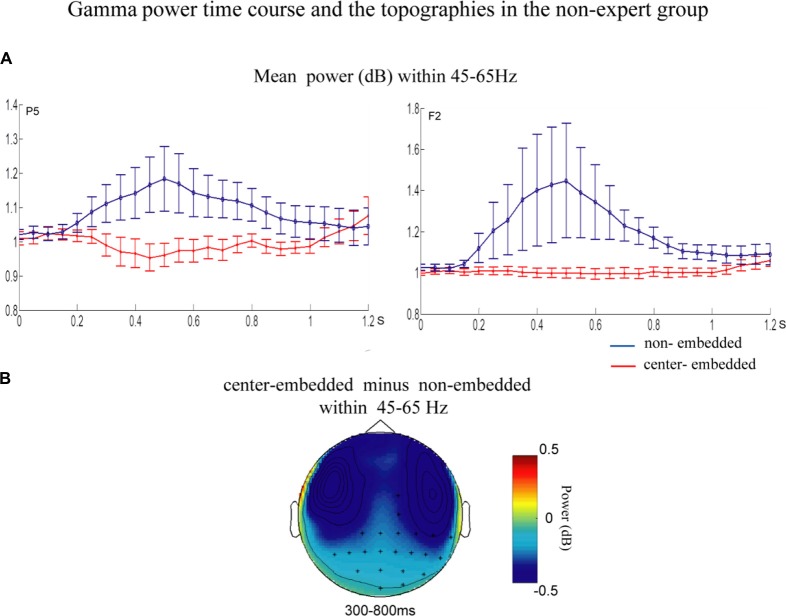
Time course of mean gamma power and difference topographies of final chords in the non-expert group.** (A)** Temporal course of the mean gamma power (45–65 Hz) between 0 ms and 1200 ms after the onset of the final chords for the non-embedded and center-embedded conditions at electrodes P5 and F2. Error bars indicate standard errors. **(B)** The difference topographies (center-embedded minus embedded) in the gamma band (45–65 Hz) within 300–800 ms. The electrodes that showed significant effects over 30% of the selected time intervals are marked by asterisks.

The statistical analyses for the TF data were conducted independently for the two groups. In the non-expert group, the permutation test over the entire low-frequency band (2–30 Hz) within the whole epoch (0–1,200 ms) revealed one significant cluster (*p* = 0.014) in the alpha band, suggesting that the center-embedded condition, compared to the non-embedded condition, induced an alpha power decrease (around 8–12 Hz, within around 100–600 ms, with left temporal-parietal and right frontal-parietal scalp distributions, *p* = 0.004). Figure 3 presents the TF representations of the two conditions (non-embedded and center-embedded) as well as the contrasts between them (center-embedded minus non-embedded) at electrodes P5 and F2. Figure 4 illustrates the time course of the evolution of the mean alpha frequency interval (8–12 Hz) after the onset of the final chords at electrodes P5 and F2; and the difference scalp topographies in the selected time ranges. The permutation test over the entire high-frequency band (30–100 Hz) within the whole epoch (0–1,200 ms) revealed one significant cluster (*p* = 0.018), suggesting that the center-embedded condition induced a gamma power decrease (around 45–65 Hz, within around 300–800 ms, with a parietal- occipital scalp distribution, *p* = 0.002). Figure 3 shows the TF representations of each pair of conditions (center-embedded and non-embedded) as well as the contrasts between them (center-embedded minus non-embedded) at electrodes P5 and F2. Figure 5 illustrates the time course of the evolution of the mean gamma frequency interval (45–65 Hz) after the onset of the final chords in the two conditions at electrodes P5 and F2; and the difference scalp topographies (center-embedded minus non-embedded) in the relevant time ranges.

**Figure 6:**
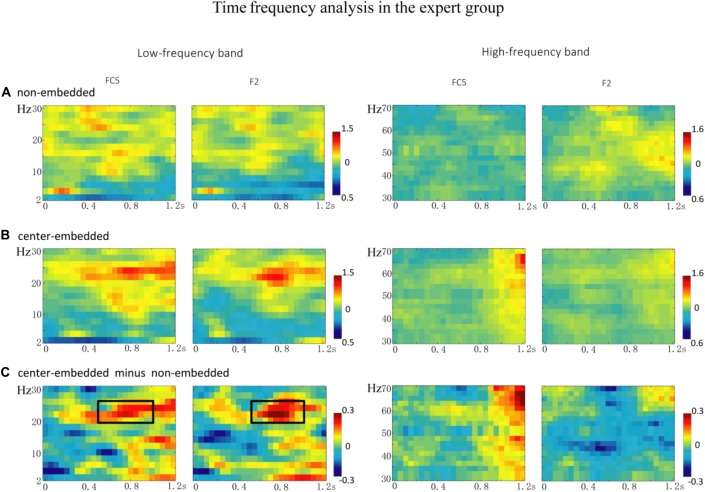
The time frequency analysis of final chords in the expert group.** (A,B)** Time frequency representations of the non-embedded and center-embedded conditions at electrodes FC5 and F2, for the low and high frequency ranges. **(C)** The contrasts of the center-embedded and non-embedded conditions at the same electrodes. The black boxes indicate the frequency-band (20–26 Hz) within the selected time window (500–1,000 ms), indicating a significant difference between the two conditions.

**Figure 7:**
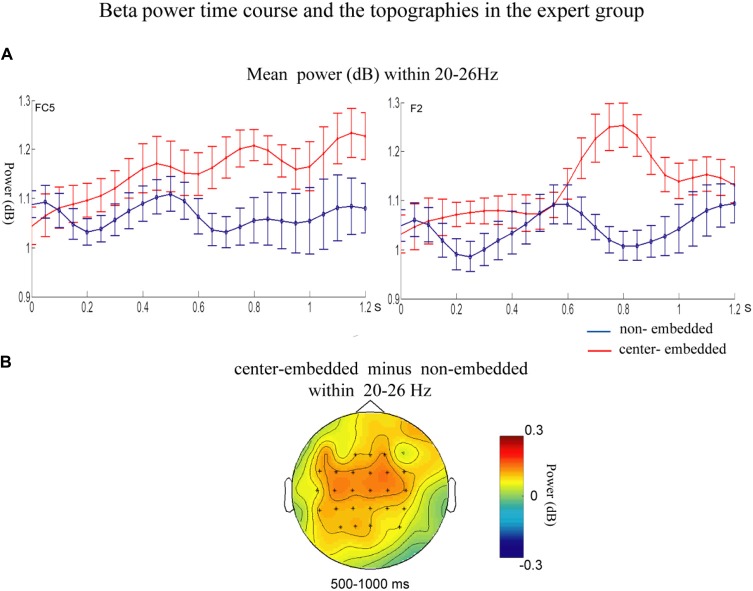
Time course of the mean beta power and difference topographies of final chords in the expert group.** (A)** Temporal course of the mean beta power (20–26 Hz) between 0 ms and 1,200 ms after the onset of the final chords for the non-embedded and center-embedded conditions at electrodes FC5 and F2. Error bars indicate standard errors. **(B)** The difference topographies (center-embedded minus embedded) in the beta band (20–26 Hz) within 500–1,000 ms. The electrodes that showed significant effects over 30% of the selected time intervals were marked by asterisks.

In the expert group, the permutation test over the entire low-frequency band (2–30 Hz) within the whole epoch (0–1,200 ms) revealed one significant cluster (*p* = 0.014), suggesting that, relative to the non-embedded condition, the center-embedded condition induced a beta power increase (around 20–26 Hz, within around 500–1,000 ms, with a frontal-parietal scalp distribution, *p* = 0.002). Figure 6 presents the TF representations of the non-embedded and center-embedded structures as well as the contrasts between them (center-embedded minus non-embedded) at electrodes FC5 and F2. Figure 7 presents the time course of the evolution of the mean beta frequency interval (20–26 Hz) after the onset of the final chords in the two conditions at electrodes FC5 and F2; and the difference scalp topographies (center-embedded minus non-embedded) in the selected time window. The permutation test over the entire high-frequency band (30–100 Hz) within the whole epoch (0–1,200 ms) revealed no significant difference between the center-embedded and non-embedded conditions (*p* = 0.399).

## Discussion

The present study investigated the differences in the neural responses to musical sequences with different syntactic structures, i.e., non-embedded and center-embedded structures, and how these neural responses were affected by music proficiency. In the non-expert group, the final chords in center-embedded sequences compared to those in non-embedded sequences elicited greater ERAN and N5 components. Furthermore, the final chords in center-embedded sequences induced alpha power and gamma power decreases. However, in the expert group, no significant differences in ERP responses to final chords were observed for the two types of sequences. Nevertheless, compared to those in non-embedded sequences, the final chords in center-embedded sequences elicited amplified of beta activity.

### Processing of the Two Types of Syntactic Sequences in the Non-expert Group

The ERAN and N5 component have been taken as the indexes of the degree of consistency of an incoming chord to the contextual syntactic constraint (Koelsch et al., 2000; Poulin-Charronnat et al., 2006; Koelsch and Jentschke, 2009; Koelsch et al., 2013). In the non-expert group, the processing of the final chords in center-embedded sequences evoked greater ERAN and N5 components than that in non-embedded conditions, showing that for lower-proficiency listeners, the difficulty in embedded structure processing was significant, and the effects appeared in both the early (a larger ERAN) and late processing stages (a larger N5). Furthermore, it indicated that the embedded segment (transposition or modulation transition) may induce a larger interference with the construction of long-distance dependency, hindering the syntactic associative access of the upper-level phrase. These results are consistent with the findings of previous psycholinguistic studies, showing that, the subordinate clause (embedded structure) can interfere with the syntactic attachment of the separate main clause. For example, compared to the processing of verbs in simple sentences (non-embedded sentences), the processing of verbs in main matrix clause in sentences with center-embedded structure tends to exhibit slower reaction times (Stromswold et al., 1996), lower accuracy rate (Opitz and Friederici, 2007) and elicit larger LAN or P600 (Vos et al., 2001; Phillips et al., 2005; Li and Zhou, 2010).

Compared to that in non-embedded sequences, the processing of final chords in center-embedded sequences evoked a power decrease in the alpha frequency band (around 8–12 Hz, mainly in the left temporal-parietal and right frontal-parietal regions) in the 100–600 ms time range. Many studies found that expectancy-deviation can lead to a decrease in the alpha power either in language sentences (Luo et al., 2010; Wang et al., 2012a; Lewis et al., 2016a) and in musical sequences (Ruiz et al., 2009; Akrami and Moghimi, 2017). Researchers consider that alpha band activity, especially in parietal and prefrontal region, is generally associated with a cognitive control activity (a type of general cognitive resource), by mediating the integration from multiple cortical areas that are activated during the task (Ruiz et al., 2009; Meyer et al., 2013; Sadaghiani and Kleinschmidt, 2016), the violation condition generally captures more cognitive control resources (e.g., selective attention and sustained alertness), which will results in desynchronization of alpha frequency activity (Wang et al., 2012a; Wilsch et al., 2015; Rommers et al., 2017). Therefore, the alpha power decrease observed in our study may be ascribed to additional demand for cognitive control and more general cognitive resources required by the complexity of recursive or embedded structure processing in music. This explanation and finding could gain support from previous studies showing that more anterior region of the PFC (e.g., BA 10/47) activated by the increased level of embeddedness (Badre, 2008; Makuuchi and Friederici, 2013; Jeon and Friederici, 2015), which also demonstrated that recursion processing, especially in low-proficiency stimuli, would recruit more general cognitive mechanism (Jeon and Friederici, 2013, 2015; Friederici and Singer, 2015).

Additionally, compared to those in non-embedded sequences, the final chords in center-embedded sequences induced a decrease in the gamma power activity (around 45–65 Hz) in the mid-term and late time windows (around 300–800 ms). Oscillatory activity in the low and middle gamma band (30–75 Hz) generally reflects the predictability of an upcoming word based on the preceding context (Wang et al., 2012b; Lewis and Bastiaansen, 2015; Lewis et al., 2015). It is considered that a lower degree of matching between the expectation and the bottom-up linguistic inputs would not lead to pre-activation or gamma power increase (or even a decrease; Hald et al., 2006; Wang et al., 2012b; Molinaro et al., 2013; Lewis and Bastiaansen, 2015; Lewis et al., 2015). Ruiz et al. (2009) provided the evidence that the lower expectations of supertonic chord evoked the gamma decrease at the frontal and parietal brain areas. Following this consideration, our results could be explained by the final chords of the center-embedded sequences may cause a lower degree of matching with the previous chord context, compared to that in the sequences without a key change in the non-embedded conditions. Thus, the lower predictability in the embedded sequences may lead to a decrease in gamma band activity.

### Processing of the Two Types of Syntactic Sequences in the Expert Group

For the expert group, there was no significant difference in the ERPs of final chords between non-embedded and center-embedded conditions indicating little difference between the processing of the two syntactic structures (although a tendency of a greater late negativity was observed during the processing of the embedded structure in the left-frontal region, see Figure 2, at electrode F1, this effect was not significant), Similarly, the findings of psycholinguistic studies indicate that for high-proficiency learners, increased syntactic complexity does not necessarily lead to greater processing difficulty (King and Kutas, 1995; Jackson and van Hell, 2011). Researchers suggested that increase processing proficiency, may decrease the effort cost or difficulty and in complex sentence processing (Clahsen and Felser, 2006; Jackson and van Hell, 2011; Jeon and Friederici, 2013, 2015).

Nevertheless, significant differences were observed in the neural oscillations from the processing of the final chords in the two syntactic sequences. Compared to that in simple sequences, the processing of the final chords in complex embedded sequences elicited amplified beta activity (around 20–26 Hz, with a frontal-parietal scalp distribution) in the time window of 500–1,000 ms. Studies in the field of language have found that increased structure complexity typically induces higher beta power (Weiss et al., 2005; Bastiaansen and Hagoort, 2006; Meyer et al., 2013; Schneider et al., 2016). Researchers have proposed that higher beta power, especially in frontal region is related to active maintenance of the current cognitive set with regard to the current sentence-level structural or semantic representation under construction (Wang et al., 2012a; Bressler and Richter, 2015; Lewis and Bastiaansen, 2015; Lewis et al., 2015, 2016b). Therefore, when syntactic processing becomes more challenging or the demand on syntactic unification increases in more complex sentences, which suggests the current mode of processing (unification) needs to be actively maintained (Lewis and Bastiaansen, 2015; Lewis et al., 2015, 2016b), so the beta power will increase (Bastiaansen and Hagoort, 2006; Meyer et al., 2013; Lewis et al., 2015; Akrami and Moghimi, 2017). In our study, compared to non-embedded structure, the beta power increase elicited by center-embedded structure processing may be explained in similar ways. For the expert group equipped with knowledge of the two structures, however, the processing of more abstract and complicated grammar in embedded or recursive structures (the more difficult in syntactic computation) will increase the need to maintain unification operations relying on top-down predictions. This is in line with the findings of previous psycholinguistic studies, showing that the processing center-embedded structure usually elicit larger beta power or enhanced activities in BA44 with increased level of embeddedness (Opitz and Friederici, 2007; Makuuchi et al., 2009; Jeon and Friederici, 2013), indicating the processing of center-embedded structure increased the operation of unification that referring to reanalysis and reconstruction (Friederici, 2006, 2011; Brouwer and Hoeks, 2013).

### Differences Between Musical Syntactic Processing in the Expert and Non-expert Groups

We found significant differences in ERP responses between the experts and the non-experts. The processing difficulties arising from increased syntactic complexities were prominent in the non-experts, but not in the experts, indicating that the non-experts have greater difficulty in processing center-embedded sequences than the experts. This result seems to be inconsistent with the finding of Koelsch et al. (2013), in which no difference was found between experts and non-experts in the processing of center-embedded sequences. We consider that the discrepancy could partly be ascribed to the participants, non-native listeners (Chinese) of Western music used in our study, whereas native listeners (German) in their study. Data from bimusicalism studies have revealed that compared to the native learners, non-native learners showed very limited musical competence if lack of professional training (Demorest and Osterhout, 2012; Demorest et al., 2016). For increased syntactic complexity, non-native listeners have to pay higher cognitive cost and still show a larger gap with the high-proficiency individuals (Matsunaga et al., 2014). In contrast, the native music learners can have higher competence in syntactic processing even without professional training because of their long term exposure to western music (Koelsch et al., 2013), and show a smaller gap with the high-proficiency individuals.”

The significant interactions between structural complexity and musical proficiency were observed in the neural oscillations, indicating that different strategies were adopted by experts and non-experts in order to process complex recursive or embedded structures. In the expert group, the final chords in center-embedded sequences induced a beta power increase compared to those in non-embedded sequence, reflecting the greater demand on syntactic unification (Lewis and Bastiaansen, 2015; Lewis et al., 2015, 2016b). That is, to deal with the increased of the syntactic complexity, the expert group might increase the dependance on maintenance of unification based on deep tonal structure representation.

Unlike the expert group, however, the non-expert group did not show the beta effects, suggesting that this group might have more used other processing mechanisms as reflected by the significantly reduced alpha and gamma band activity. Alpha activity may reflect involvement of general cognitive mechanisms (especially relate to cognitive control; Meyer et al., 2013; Sadaghiani and Kleinschmidt, 2016). Besides, the result showing gamma activity without beta activity might be interpreted as a “shallow” mode of syntactic parsing (Sanford and Sturt, 2002; Clahsen and Felser, 2006; Lewis et al., 2016a). According to Memory, Unification and Control model (MUC; Hagoort, 2014) and Retrieval-Integration account (Brouwer et al., 2012; Brouwer and Hoeks, 2013), two cognitive processes play a crucial role during language comprehension: retrieval (activation) of incoming stimulus information from memory, and unification of this incoming stimulus information into the deep representation of overall sentence. The predictability reflected by gamma activity more refer to the retrieval (activation) process (Lewis et al., 2016b; Lewis and Bastiaansen, 2015), and the retrieval process has been considered as an preliminary and shallow parsing process which the difficulty of retrieval could be determined by surface cues as the retrieval is facilitated if the some features of an incoming stimulus are consistent with the features already activated by the preceding context (Brouwer et al., 2012; Brouwer and Hoeks, 2013). In our study, the processing of more complex center-embedded syntax elicited gamma activity but less beta activity in the non-experts, implicating that the processing might be more involved in the retrieval (activation) process which is surface or shallow, but might be little involved in the deep unification process that can be reflected by beta activity. We speculated that perhaps because the non-experts more relied on such “surface” or “shallow” mode of syntactic parsing (syntactic construction more relied on surface structure), while making little use of deep syntactic parsing (Sanford and Sturt, 2002; Clahsen and Felser, 2006; Skeide et al., 2014; Lewis et al., 2016a), thus they were more disturbed by the surface structure (the embedded modulation/transitions), resulting in more difficulty in final chords processing of center-embedded sequences (as less-expectation). Notably, the expert group exhibited a similar tendency, as reflected by the gamma-power decrease during the center-embedded sequence processing compared to non-embedded sequence processing, although the decrease was not significant. This may indicate that the expert group also elicited the surface syntactic parsing during the complicated syntax processing, but to a lesser extent than the non-expert group. However, deep syntactic parsing may have increased simultaneously, as reflected by the beta power activity.

Overall, our study should be considered as a preliminary and initiative attempt to explore the neuro-cognitive mechanisms underlying center-embedded structure processing in music. It is found that there are some commonalities in such complex structure processing among different cognitive domains (music and language). As reflected by the activity of different neural oscillations, various neurocognitive mechanisms will engage in musical syntax processing with the embeddedness added. However, listeners at different proficiency level may deal with the increase of syntactic complexity by virtue of different cognitive neural mechanisms. We tentatively propose that higher-proficiency listeners are more likely to increase the dependence on unification based on top-down processing, while lower-proficiency listeners may rely on or turn to more general cognitive mechanisms (e.g., cognitive control). Future research with additional techniques, such as MEG, need to further clarify these findings as the precise brain regions involved in time-frequency activation can hardly be observed with the current EEG technology, therefore it is difficult to accurately reveal the neural mechanisms underlying the musical structure processing.

## Conclusion

We found that, when listening to musical sequences following two kinds of syntactic structure, non-embedded and center-embedded, the processing of center-embedded structure elicited larger ERAN and N5 components than the non-embedded structure in the non-expert group, while the two structures elicited indistinguishable ERP responses in the expert group. With regard to neural oscillations, the processing of center-embedded structure elicited reduced alpha and gamma power activities for the non-expert group, and increased beta activity in the expert group. Our findings expand upon the results of previous studies in language domains, and also demonstrate significant interactions between structural complexity and proficiency during musical syntax processing. The results indicate that the neurocognitive mechanisms involved in processing center-embedded structure are distinct from those involved in processing non-embedded structure in music; however, the listeners with different proficiency would dependent on different neurocognitive mechanisms in music processing as the syntactic complexity increases.

## Author Contributions

XM and YY proposed the experiment and designed the procedure, did the most work of the article. ND gave useful comments to the experiment and helped to revise the article for several times. YT helped to find participants and did the experiment work.

## Conflict of Interest Statement

The authors declare that the research was conducted in the absence of any commercial or financial relationships that could be construed as a potential conflict of interest.
