# Supplementary material for: Differences in Neurocognitive Mechanisms Underlying the Processing of Center-Embedded and Non–embedded Musical Structures
Source: Front Hum Neurosci. 2018 Oct 23;12:425. doi: 10.3389/fnhum.2018.00425 (PMC6206303; doi:10.3389/fnhum.2018.00425)
Supplement: Supplementary file 1 [file Data_Sheet_1.docx]

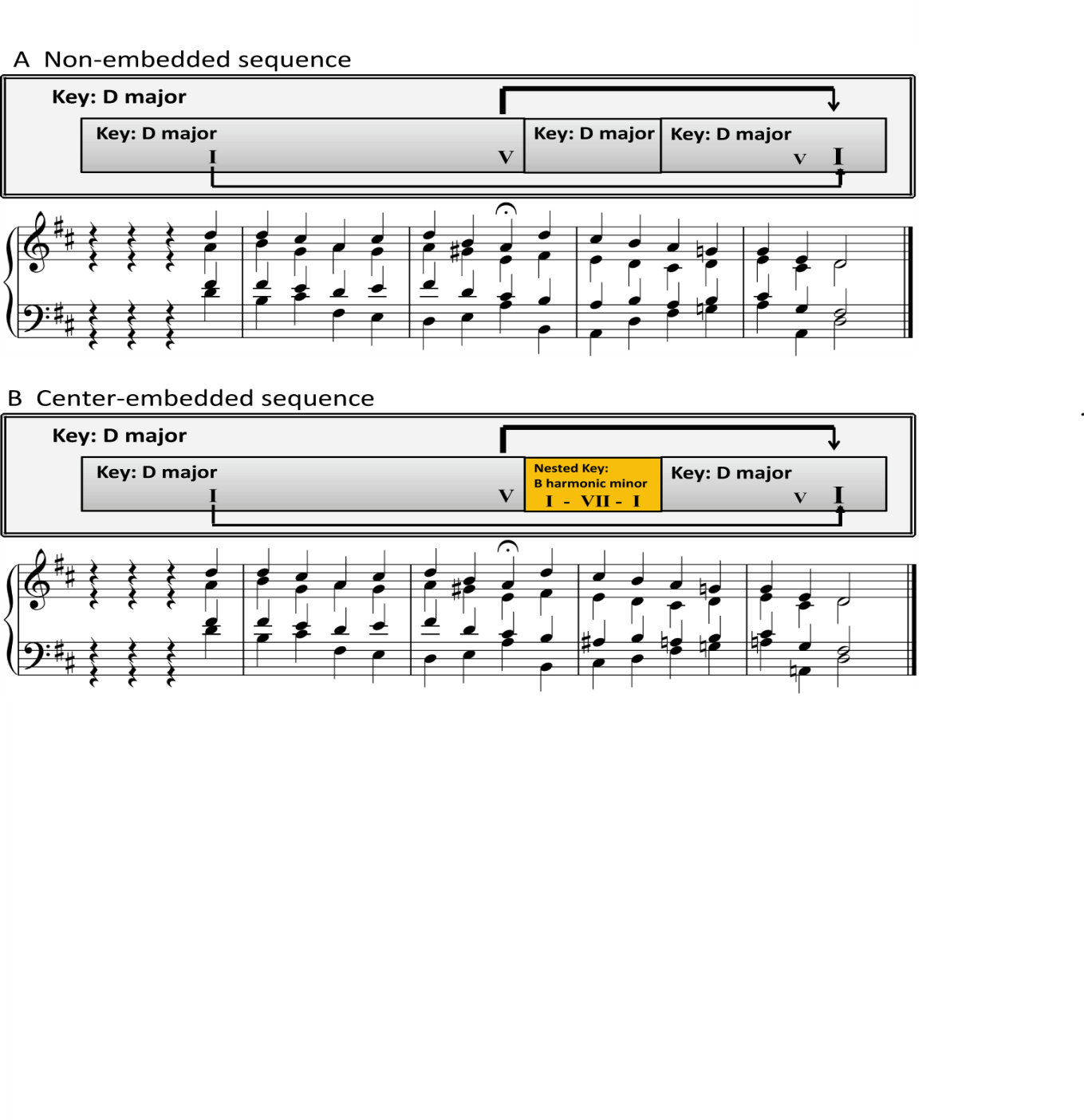


**Fig.S1. A sample excerpt illustrates the two sequences with different structure.**

A: The non-embedded sequences, chord sequences without transition or modulation segments. All parts are in D major. B: The center-embedded sequences, chord sequences with transitions modulation segments, the B harmonic minor segment is nested into an overarching key of D major,
